# ATP and Adenosine in the Retina and Retinal Diseases

**DOI:** 10.3389/fphar.2021.654445

**Published:** 2021-06-15

**Authors:** Shan-Shan Ye, Yong Tang, Jian-Tao Song

**Affiliations:** ^1^ Eye Hospital, China Academy of Chinese Medical Sciences, Beijing, China; ^2^ International Collaborative Centre on Big Science Plan for Purinergic Signalling, Chengdu University of Traditional Chinese Medicine, Chengdu, China; ^3^ Acupuncture and Chronobiology Key Laboratory of Sichuan Province, Chengdu, China

**Keywords:** ATP, adenosine, age-related macular degeneration, glaucoma, diabetic retinopathy

## Abstract

Extracellular ATP and its ultimate degradation product adenosine are potent extracellular signaling molecules that elicit a variety of pathophysiological pathways in retina through the activation of P2 and P1 purinoceptors, respectively. Excessive build-up of extracellular ATP accelerates pathologic responses in retinal diseases, whereas accumulation of adenosine protects retinal cells against degeneration or inflammation. This mini-review focuses on the roles of ATP and adenosine in three types of blinding diseases including age-related macular degeneration (AMD), glaucoma, and diabetic retinopathy (DR). Several agonists and antagonists of ATP receptors and adenosine receptors (ARs) have been developed for the potential treatment of glaucoma, DR and AMD: antagonists of P2X7 receptor (P2X7R) (BBG, MRS2540) prevent ATP-induced neuronal apoptosis in glaucoma, DR, and AMD; A1 receptor (A1R) agonists (INO-8875) lower intraocular pressure in glaucoma; A2A receptor (A2AR) agonists (CGS21680) or antagonists (SCH58261, ZM241385) reduce neuroinflammation in glaucoma, DR, and AMD; A3 receptor (A3R) agonists (2-Cl-lB-MECA, MRS3558) protect retinal ganglion cells (RGCs) from apoptosis in glaucoma.

## Introduction

**FIGURE 1:**
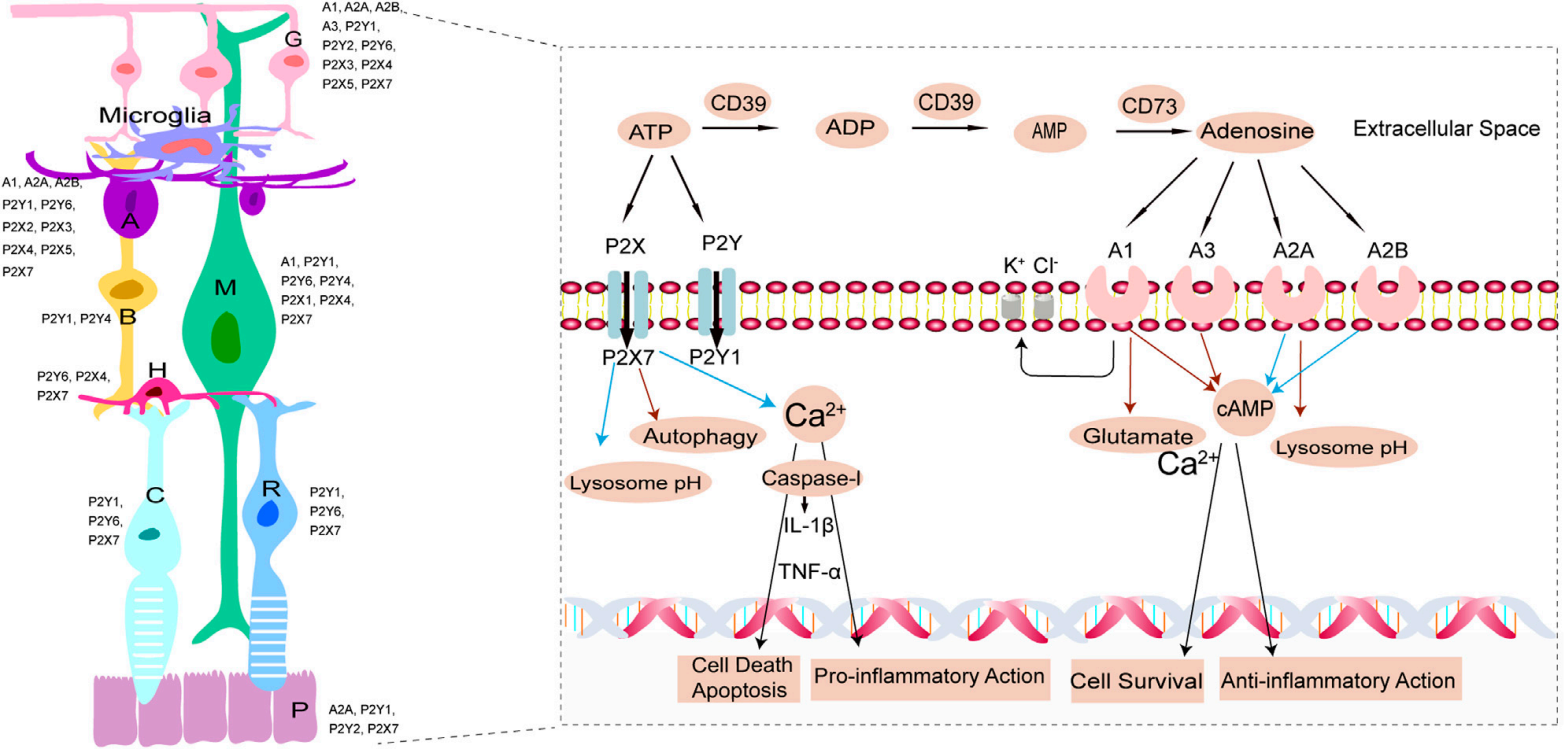
In the left, schematic representation illustrating the expression of P2 and P1 receptors (Ventura et al., 2019). *P*, Pigment epithelium cell; R, rod cell; C, cone cell; H, horizontal cell; B, bipolar cell; M, Müller cell; A, amacrine cell; G, ganglion cell. In the right, ATP and adenosine metabolizing in the extracellular conditions and mediate pathologic process within the retina. Extracellular ATP is hydrolyzed to ADP, AMP, adenosine by the activity of CD39 and CD73. Extracellular ATP and adenosine interact, therefore causing their activation, with P2 (P2X, P2Y) and P1 (A1, A2A, A2B, A3) purinergic receptors, respectively. On one hand, Calcium-dependent cell death pathways due to increased ATP or glutamate and inflammasome-dependent cell death pathway induced by P2X7 pore formation accompanied with activation of apoptosis-inducing factor, both contribute to retinal cells death or apoptosis. Extracellular ATP stimulates retinal microglia activity to release neurotoxic factors, (e.g.TNF-α, IL-1β) exerting pro-inflammatory action. Stimulation of P2X7R raises lysosomal pH (blue arrows) in RPE cells and lowers autophagy, which contribute to lysosomal alkalinization and lipofuscin accumulation. On the other hand, A1R and A3R inhibit adenylyl cyclase decreasing intracellular cyclic adenosine monophosphate (cAMP) levels (red arrows) while A2AR and A2BR activate adenylyl cyclase increasing intracellular cAMP levels (blue arrows). Adenosine acting at A1R significantly decreases the glutamate-induced calcium influx (red arrows) contributing to cell survival. Adenosine exerts anti-inflammatory action, as well as decreases lysosome pH (red arrows). Moreover, A1R opening the potassium and chloride channels, increases the fluid clearance from the edematous retina via increased extrusion into the blood of ionic osmolytes.

Purines and their derivatives, most notably adenosine and ATP, are the key molecules controlling intracellular energy homoeostasis and nucleotide synthesis (Huang et al., 2021). High concentrations of ATP are present within cells and ATP is released into the extracellular milieu during cellular damage or death. Extracellular ATP acts at P2 receptors, including ligand-gated ion channel (P2X1-7) receptors and metabotropic G-protein linked purinergic receptors (P2Y1,2,4,6,11-14) (Jacobson et al., 2020; Illes et al., 2021), whereas adenosine acts at P1 G protein-coupled receptors, classified into four subtypes: A1R, A2AR, A2BR and A3R (Santiago et al., 2020). Expression of several P1 and P2 receptors were detected in the retina (Figure 1) (Ventura et al., 2019). ATP and adenosine metabolizing in the extracellular conditions mediate pathologic process within the retina (Figure 1). ATP signaling is tightly regulated by ectonucleotidases including ectonucleoside triphosphate diphosphohydrolases (NTPDases) such as CD39, which rapidly hydrolyses ATP and ADP to AMP (Dwyer et al., 2020). And then 5′-ectonucleotidase CD73 degrades AMP to adenosine (Dwyer et al., 2020). Commonly, ATP and adenosine acting at their respective receptors generate opposite responses (Kukulski et al., 2011; Sanderson et al., 2014; Jacobson et al., 2020; Illes et al., 2021). Excessive extracellular ATP released from stressed retinal cells is recognized as an endogenous danger signal in retinal injury or age-related macular degeneration (AMD), glaucoma, and diabetic retinopathy (DR) (Notomi et al., 2011; Niyadurupola et al., 2013). In this review, we focused on the mutual or opposite roles of ATP and adenosine in retinal cells linked to the three types of blinding diseases (AMD, glaucoma, and DR), as well as emphasized the therapeutic potential of agonists and antagonists of ATP receptors and ARs for these diseases.

## ATP and Adenosine in the Retina

### ATP and Adenosine in the Retinal Neuronal Cell Death or Survival

ATP is released from all main types of retinal neurons (horizontal, bipolar, amacrine, and ganglion cells) being a co-transmitter with inhibitory (GABA), or excitatory (glutamate) neurotransmitters, and neuropeptides (Ward et al., 2010). Similar to glutamate excitotoxicity, high levels of extracellular ATP released by stressed cells activate P2 receptors on neighboring neurons causing influx of calcium. The cytotoxic calcium overload is induced by overactivation of P2X receptors evoking calcium influx through receptors themselves or through voltage-gated calcium channels and by activation of P2Y receptors that induces a sustained calcium influx following rapid transient release of calcium from internal stores (Fletcher, 2010; Aplin et al., 2014; Fowler et al., 2014). Much attention has been attracted to P2X7R which forms large plasma membrane pores that mediate cytolysis and inflammasome-dependent cell death (Fowler et al., 2014). Under pathological conditions, the upregulation of P2X7R predisposes retinal neurons to damage. For instance, the increased level of extracellular ATP caused by the elevation of the intraocular pressure in glaucomatous eyes chronically activates P2X7R thus leading to death of a subpopulation of RGCs (Zhang et al., 2007). Apart from the ATP released from the retinal cells to cause cell death, exogenous administration of ATP also impairs retinal cells (Puthussery and Fletcher, 2009). Further evidence demonstrated that the BzATP- (P2X7R agonists)-mediated Ca^2+^ increases is 112-fold higher compared to ATP in cultured RGCs, when both agonists were used at 10 µM; 62-fold at 30 μM; and 23-fold at 100 µM. BzATP led to a concentration-dependent reduction in the number of cells with a median lethal dose of 35 µM, which was prevented by the P2X7R antagonists BBG and oxidized ATP, rather than 30 µM suramin (a nonselective P2 receptor antagonist) (Zhang et al., 2005). Moreover, high concentrations of ATP injected into the eyes of rodents (Puthussery and Fletcher, 2009) or feline (Aplin et al., 2014) model also lead to rapid irreversible loss of photoreceptor. Intravitreal injection of 50 mM ATP causes significant loss of visual function within 1 day and loss of 50% of photoreceptor cell within 1 week in eyes of rats (Vessey et al., 2014). Thirty hours after 100 μL intravitreal injection of ATP with a concentration of 55 mM in a feline model, widespread photoreceptor cell death took place (Aplin et al., 2014); and injection of ATP with concentrations of 11, 22, or 55 mM caused loss of retinal function and gross changes in retinal structure within 2 weeks.

Adenosine protects neurons against hyperexcitation and glutamate toxicity by inhibiting presynaptic voltage-dependent calcium channels thus reducing transmitter release of glutamate, acetylcholine, and ATP (Chen et al., 2014; Santos-Rodrigues et al., 2015), or inhibiting N-methyl-d-aspartate (NMDA) receptors (Hartwick et al., 2004). Adenosine modulates RGC function and confers general neuroprotection through A_1_R (Lu et al., 2017). Acting at A3R adenosine inhibits P2X7-induced increases in calcium and apoptosis of RGCs (Boia et al., 2020). In isolated rat RGCs, adenosine (10 and 100 μM) significantly decreased the glutamate-induced calcium influx, which was blocked by A1R antagonist 8-cyclopentyl-1,3-dipropylxanthine (DPCPX) (Hartwick et al., 2004). Chronic activation of A2AR dependent cAMP signaling prevents glutamate-induced cell death in chick retinal neurons and photoreceptors (Paes-de-Carvalho et al., 2003). Stimulation of A3R by either endogenous or synthesized agonist (2‐Cl‐lB‐MECA or MRS3558) can reduce calcium response to NMDA receptor activation in retinal ganglion cells (Zhang et al., 2010). These data suggest that the adenosine exerts an inhibitory influence on retinal neurons by modulating excitatory glutaminergic signaling, thus promoting retinal neural cells survival. Stimulation of A1R with the agonist N-Cyclopentiladenosine (CPA) protected against photoreceptor cell death in light-induced retinal degeneration model probably due to the presynaptic inhibition of glutamate release and the modulation of NMDA receptor activity (Soliño et al., 2018). This protective capacity of adenosine at photoreceptor cell is consistent with neuromodulator role of adenosine in glutaminergic signaling.

### ATP and Adenosine in the Retinal Pigment Epithelium (RPE) Cell Degeneration

ATP can be released at sites of inflammation and upregulate P2X7R expression in human epithelial cells; P2X7R mRNA and protein are also expressed in retinal pigment epithelium (RPE) cells (Yang et al., 2011). Therefore, ATP released during pathologic conditions increases P2X7 expression in the RPE and thus increases the vulnerability of RPE cells to extracellular ATP-induced cell death. Both endogenous P2X7 agonist ATP and the synthetic, P2X7 agonist BzATP increased intracellular Ca^2+^ by extracellular Ca^2+^ influx and induced apoptosis of RPE cells. These effects are inhibited by P2X7 antagonist OxATP but not by the P2 receptor antagonist suramin (Yang et al., 2011), indicating that ATP induced RPE apoptosis by activation of P2X7R. Stimulation of P2X7 also activates p38, which induces secretion of monocyte chemoattractant protein-1 (MCP-1), interleukin 8 (IL-8), and vascular endothelial growth factor (VEGF); IL-8 and VEGF promote angiogenesis leading to choroidal neovascularization, a hallmark of wet AMD (Fowler et al., 2014). Balance between extracellular ATP and adenosine alters lysosomal activity of RPE cells and the production of lipofuscin. Stimulation of P2X7R raises lysosomal pH in RPE cells and decreases autophagy, which contributes to lysosomal alkalinization (Guha et al., 2013) and digestion impairment of peroxidized photoreceptor lipids, resulting in accumulation of lipofuscin and formation of lipoprotein-containing drusen beneath the RPE (Guha et al., 2013). Conversely, adenosine stimulating A2AR reacidifies lysosomes. The mechanism underlying restoration of acidic lysosomal pH may be linked to the elevation of cytoplasmic cAMP following stimulation of adenosine A2AR (Liu et al., 2008). In summary, elevation of adenosine signaling represents a positive response of RPE cells to AMD.

### ATP and Adenosine in the Müller Cell Volume

Müller cells intimately contact neurons and non-neural structures providing for uptake of neurotransmitters from extracellular space, as well as controlling retinal potassium and water homeostasis to maintain the extracellular space volume (Bringmann et al., 2006). Activation of P2X7 depolarizes Müller cells, thus decreasing the rate of the glial glutamate uptake resulting in elevated extracellular glutamate levels. Glutamatergic neurotransmission evokes ion currents which lead to osmotic imbalances and a swelling of neuronal cells bodies and synapses (Vogler et al., 2013). Intense neuronal activity also decreases extracellular osmolarity due to the activity-dependent decrease in extracellular sodium chloride which is approximately twice as large as the increase in extracellular potassium (Vogler et al., 2013). Then, decreased extracellular osmolarity and the uptake of neuron-derived osmolytes contribute to Müller cells swelling and retinal edema (Vogler et al., 2013). Under this hypoosmotic conditions, passive potassium efflux through Kir4.1 channels and activation of a purinergic signal transduction cascade (consecutive ATP and adenosine from Müller cells) are two main mechanisms preventing swelling of Müller cells (Pannicke et al., 2004). Release of ATP from Müller cells is central to maintain water dynamics in the retina, while preventing this release causes retinal swelling and edema (Reichenbach and Bringmann, 2016). On the other hand, adenosine acting at A1R results in the opening of potassium and chloride channels that allow ion efflux thus increasing fluid clearance from the edematous retina (Schwartz et al., 2016).

Osmo-mechanosensitive release of ATP from Müller cells is impaired in diabetic retinopathy (DR). In contrast, glutamate-induced release of ATP from Müller cells remains protective against osmotic swelling. Various receptor ligands such as vascular endothelial growth factor (VEGF) induce a release of glutamate from Müller cells thus contributing to purinergic signaling involved in the inhibition of osmotic swelling (like above) (Reichenbach and Bringmann, 2016; Schwartz et al., 2016). Extracellular ATP is converted by NTPDase2 to ADP that activates P2Y1, further facilitating release of adenosine by nucleoside transporters and ultimately inhibit cell swelling (Weick et al., 2005). These data suggest that extracellular ATP contributes to neuronal hyperexcitation and edema development under pathological conditions, whereas VEGF-induced glutamate and glutamatergic-purinergic signaling (especially adenosine signaling) prevent retinal edema. Therefore, activation of P2Y1R or A1R would be considered as a potential strategy for the control of retinal edema.

### ATP and Adenosine in the Microglial Activity

Microglia contribute to retinal inflammation, migrating toward the region of injury where they phagocyte pathogens. However, excessive or prolonged activation of microglial reactivity can cause chronic microgliosis and loss of autoregulatory mechanisms thus leading to retinal inflammation and degeneration (Kerur et al., 2013; Santiago et al., 2020). Extracellular ATP stimulates retinal microgliosis in response to overstimulated glutamatergic transmission which is a pathogenetic factor in hypoxia, glaucoma, DR, and photoreceptor degeneration (Liu et al., 2016; Loukovaara et al., 2017). Reactive microglia promote the release of neurotoxic factors such as tumor necrosis factor-α (TNF-α), IL-1β, oxygen and nitrogen free radicals, and Fas-ligand, contributing to neuronal and photoreceptor cell degeneration (Baudouin et al., 2020). Upregulation of TNF-α, IL-1β, and IL-6 that occurs following P2X7 activation induces RGCs death under elevated intraocular pressure (Sugiyama et al., 2013). Therefore, suppression of microglia reactivity has protective effects.

Adenosine possesses anti-inflammatory properties in retina (Aires et al., 2019a; Aires et al., 2019b). The increased adenosine at inflamed sites can protect against cellular damage by activating A2AR (Ibrahim et al., 2011), by inhibiting release of TNF-α from microglia induced by hypoxia and lipopolysaccharide, and by inhibiting microgliosis (Jamwal et al., 2019). Cannabidiol, an anti-inflammatory molecule, prevents the adenosine uptake and subsequently activates adenosine A2AR to inhibit retinal microglia activation (Liou et al., 2008). Considering the contribution of neuroinflammation for the pathophysiology of retinal degeneration, therapies focused on pro-inflammatory ATP and immunosuppressive adenosine and their receptors are significant to retinal blinding diseases.

## Therapeutic Potential of ATP and Adenosine in Retinal Diseases

### Glaucoma

Increased vitreal concentration of ATP has been identified as a contributing factor in the death of ganglion cells and observed in animal models and humans with glaucoma (Reigada et al., 2008), the second leading cause of blindness in the world, accompanied by elevated intraocular pressure (IOP), death of RGC, and increased inflammatory response (Baudouin et al., 2020). ATP modulates retinal neurotransmission, affecting retinal blood flow and intraocular pressure. The ATP analog β,γ-methylene ATP is more effective in reducing intraocular pressure (40%) than muscarinic agonists such as pilocarpine (25%) and β-adrenoceptor blockers (30%), raising the potential for the use of purinergic agents in glaucoma (Burnstock, 2006). In chronic glaucoma, ATP is released via pannexin hemichannels in astrocytes of the optic nerve and stimulates P2X7R that can lead to cell death in the retina (Beckel et al., 2014). Antagonists of the P2X7R prevent neuronal apoptosis in the retina induced by ATP. At present, the P2X7R antagonists Brilliant Blue G (BBG) and MRS2540 have been proved to be useful in treating glaucoma (Sakamoto et al., 2015; Dong et al., 2018). Agonists of P2Y2 receptor (P2Y2R) and P2Y6 receptor (P2Y6R) decrease IOP (Markovskaya et al., 2008; Ginsburg-Shmuel et al., 2012; Jacobson and Civan, 2016). Topically applied P2Y6R agonist UDP reduced rabbit IOP by 17%, while TG46, a potent agonist of the P2Y6R, administered topically decreased IOP in rabbits by 45% (Markovskaya et al., 2008; Ginsburg-Shmuel et al., 2012).

Treatment with A1R agonists (such as INO-8875) lowers IOP by enhancing aqueous humor outflow (Lu et al., 2017). A2AR agonist CGS21680 decreased retinal inflammation in a mouse model of traumatic optic neuropathy (Ahmad et al., 2013). A3R agonists such as 2-Cl-lB-MECA (Galvao et al., 2015; Boia et al., 2020) and MRS3558 (Jacobson and Civan, 2016) protect RGCs from apoptosis induced by ATP and other factors that activate the P2X7R. Therefore, targeting this receptor might be beneficial for glaucoma affecting RGCs. Selective A_2A_R antagonist (SCH58261) suppressed elevated pressure-induced inflammation, oxidative stress, and cell death in retinal cells (Aires et al., 2019a), as well as prevented RGC death from high intraocular pressure-induced transient ischemic injury (Madeira et al., 2016). Intravitreal injection of A2AR antagonist ZM241385 reduced microgliosis and downregulated the proinflammatory cytokines expression in rat model (Liu et al., 2016). These apparent contradictory results might be explained by the type of injury inflicted, different modes of drug administration, and the cell types involved. This issue is significant because effects mediated by A2AR are bidirectional, depending on the target cell (Dai and Zhou, 2011). Another interesting study indicated that the relationship between intake of caffeine, a nonselective antagonist of ARs, and glaucoma was null, but greater consumption of caffeine was associated with higher prevalence of IOP and glaucoma (Kim et al., 2020).

### Diabetic Retinopathy

DR is another major cause of blindness, accompanied by chronic inflammation, neuronal and glial dysfunctions, and cell death. Metabolic disorders and hyperglycemia may lead to change of intracellular and extracellular nucleotide levels (Loukovaara et al., 2017). Extracellular ATP induces formation of P2X7R pores and cell death in retinal microvessels in the model of DR (Burnstock and Novak, 2013; Burnstock, 2018), suggesting that purinergic vasotoxicity plays a crucial role in microvascular cell death, a feature of DR. With respect to extracellular ADO in DR, it was demonstrated that triamcinolone, used clinically for the rapid resolution of diabetic macular edema, can induce the release and formation of endogenous adenosine and subsequently activate A1R receptors resulting in ion efflux through potassium and chloride channels and prevention of osmotic swelling (Wurm et al., 2008). A1R-dependent mechanism may contribute to the inhibition effect of triamcinolone on osmotic swelling of Müller glial cells in retinas (Uckermann et al., 2005; Wurm et al., 2008). Using A2AR agonist CGS21680 decreased hyperglycemia-induced retinal cell death, and reduced tumor necrosis factor-α release in activated microglia in DR (Ibrahim et al., 2011). Intravitreal injection of the A2AR antagonist SCH58261 protected the retina against microglial reactivity and neuroinflammation, rescued retinal vascular leakage, reduced retinal cell death and the loss of retinal ganglion cells induced by diabetes (Aires et al., 2019b).

Furthermore, caffeine could reduce RPE cells monolayer permeability after exposure to high glucose and desferoxamine, and prevent outer blood-retinal barrier (BRB) damage by inhibiting apoptotic cell death (Maugeri et al., 2017). Neuroprotective effect of caffeine on RPE cell monolayer is likely to be mediated through the antagonism of A2AR. This study provides a potential innovative drug for diabetic macular edema (DME). Nevertheless, another study carried out on chick embryo retinas showed that caffeine exposure raised A2AR expression at 18 and 24 h, while decreased A2AR expression after 48 h (Brito et al., 2016). Retinas exposed to caffeine had increased levels of phosphorylated extracellular signal-regulated kinase and cAMP-response element binding protein, but decreased protein expression of tyrosine hydroxylase, calbindin and choline acetyltransferase (Brito et al., 2016). Such modulatory role of caffeine in retinas might be directly associated with regulation of adenosine system, as well as different neurotransmitter systems. To further confirm the protective effect of caffeine is mediated by antagonism on AR, future works are needed to evaluate the caffeine’s effect on expression of AR and AR-associated downstream effector genes.

### Age-Related Macular Degeneration (AMD)

AMD is responsible for ∼9% of all cases of blindness worldwide (Stahl, 2020). The disease is characterized by pathological alteration in the RPE and loss of photoreceptor cell (Stahl, 2020). Activation of P2X7R induces extracellular Ca^2+^ influx and lysosomal alkalinization in the RPE leading to apoptosis and degeneration (Yang et al., 2011; Fletcher, 2020). In wet AMD with sub-retinal hemorrhage, the release of extracellular ATP induced severe photoreceptor cell loss and the vitreous samples of AMD patients showed higher extracellular ATP levels. All hallmarks of photoreceptor cell apoptosis were prevented by a selective P2X7R antagonist BBG (Notomi et al., 2011, 2013), encouraging the potential application of BBG as a neuroprotective agent in retinal degeneration linked to excessive extracellular ATP. Stimulation of A2AR with specific agonists CGS21680 can reacidify damaged lysosomes (Liu et al., 2008). Nevertheless, A2AR antagonist, SCH58261 decreases upregulation of the expression of pro-inflammatory mediators in human microglial cells, as well as decreases the inflammatory response, ultimately increasing the clearance of apoptotic photoreceptor cells (Madeira et al., 2018).

## Conclusion

In this review, current information on the roles of extracellular ATP and adenosine involved in the pathophysiological processes within retina demonstrated that extracellular ATP acts as a neurotransmitter while adenosine exerts an inhibitory influence on retinal neuron by modulating excitatory glutaminergic signaling. Very large concentrations of extracellular ATP cause cell death while adenosine promotes cell survival in retinal neural cells and RPE cells. What’s more, extracellular ATP and adenosine balance lysosomal activity in RPE cells, as well as Müller cells volume; pro-inflammatory ATP and immunosuppressive adenosine maintain retinal microglia activation linked to neuroinflammation and retinal degeneration. Once excessive, extracellular ATP released from retinal cells during inflammation, oxidative and osmotic stress, ischemic hypoxia, mechanical stimulation, and cell injury aggravated the development of AMD, glaucoma, and DR.

Based on the commonly harmful role of extracellular ATP and the protective role of adenosine described above, it is well established that inhibition of P2 receptors or activation of P1 receptors are possible way to treat retinal diseases. In particular, P2X7-targeted therapy protects retinal cells from degeneration and the P2X7 antagonist BBG is an approved adjuvant in ocular surgery. Selective agonists of ARs, especially A1R and A3R which prevent inflammation, neuronal hyperexcitation and P2X7 activation, are demonstrated by multiple studies *in vitro* or vivo to lower intraocular pressure, reduce neuroinflammation and apoptosis. However, conflicting data are available regarding a potential use of A2A antagonists for the protection from retinal degeneration. Beneficial or maleficent effects of caffeine, a nonselective antagonist of A2AR, have been demonstrated by different studies dependent on different time windows of caffeine response or animal development phase, even though available study proposed that caffeine could be considered as innovative therapeutic drug for macular edema.

However, what is the role of other purinergic receptors in retina and retinal diseases remains unclear. For instance, many drugs targeting purine metabolism or purinergic signaling have been developed. The US FDA database shows that several approved purinergic signaling target-related drugs, including adenosine and caffeine citrate injection, are mainly used in the cardiovascular system (Huang et al., 2021). In addition, Gefapixant (AF-219) (P2X3R antagonist) is used for the chronic cough. However, the dinucleotide Up4U (diuridine tetraphosphate), marketed Diquas (P2Y2R agonist), is the only drug used in clinical practice for ocular diseases (dry eye). Notably, P2Y12 receptor (P2Y12R) antagonists have been widely used as platelet inhibitors to prevent thromboembolism. But currently no clinical trials on the use of P2Y12 antagonists in retinal disease have been performed even though P2Y12 antagonists could be helpful to retinal microvascular disorders in DR. It is worthy of further study and discussion.

ATP and adenosine acting independently as receptor ligands, are interrelated as adenosine is a hydrolytic product of ATP by the activity of ectonucleotidases CD39 and CD73 in the retina. Right now few studies on the presence of ectonucleotidases in the retina are present in the literature, therefore deeper insights into these ectonucleotidases (CD39 and CD73) in mediating purinergic signaling and interacting with the immune system in relation to retinal pathophysiology might markedly promote our understanding in ATP and adenosine within retina and develop therapeutic modalities to treat retinal diseases.
